# Cephalopod Brains: An Overview of Current Knowledge to Facilitate Comparison With Vertebrates

**DOI:** 10.3389/fphys.2018.00952

**Published:** 2018-07-20

**Authors:** Shuichi Shigeno, Paul L. R. Andrews, Giovanna Ponte, Graziano Fiorito

**Affiliations:** ^1^Department of Biology and Evolution of Marine Organisms, Stazione Zoologica Anton Dohrn, Naples, Italy; ^2^Division of Biomedical Sciences, St. George’s University of London, London, United Kingdom

**Keywords:** octopus, cephalopod, brain, evolution, neural networks

## Abstract

Cephalopod and vertebrate neural-systems are often highlighted as a traditional example of convergent evolution. Their large brains, relative to body size, and complexity of sensory-motor systems and behavioral repertoires offer opportunities for comparative analysis. Despite various attempts, questions on how cephalopod ‘brains’ evolved and to what extent it is possible to identify a vertebrate-equivalence, assuming it exists, remain unanswered. Here, we summarize recent molecular, anatomical and developmental data to explore certain features in the neural organization of cephalopods and vertebrates to investigate to what extent an evolutionary convergence is likely. Furthermore, and based on whole body and brain axes as defined in early-stage embryos using the expression patterns of homeodomain-containing transcription factors and axonal tractography, we describe a critical analysis of cephalopod neural systems showing similarities to the cerebral cortex, thalamus, basal ganglia, midbrain, cerebellum, hypothalamus, brain stem, and spinal cord of vertebrates. Our overall aim is to promote and facilitate further, hypothesis-driven, studies of cephalopod neural systems evolution.

## Introduction

Due to shared computational and functional constraints on the evolutionary development of complex neural systems, phyletically distant animals often exhibit ‘phenotypic’ similarity in their neural organization (Farris, 2008; Roth, 2013; Wolff and Strausfeld, 2016; Shigeno, 2017). However, the origin and evolution of neural systems across animal phyla remains uncertain (Moroz, 2009; Northcutt, 2012; Holland et al., 2013; Holland, 2016). For example, centralization of nervous systems has occurred on more than five occasions during evolution (e.g., molluscs, annelids, nematodes, arthropods and chordates; see discussion in Moroz, 2009), and the acquisition of behavioral ‘capabilities’ such as the need for foraging strategies, spatial-, social- and instrumental-learning are all considered major driving forces in the evolution of complex brains and “high intelligence” several times independently in the animal kingdom (Roth, 2015). New evidence supports the view that nervous systems are not monophyletic, suggesting widespread homoplasy in nervous systems (Moroz, 2009; Liebeskind et al., 2016).

Invertebrate nervous systems are extremely diversified spanning from diffuse nerve nets (e.g., cnidarians) to tetra-neury (molluscs), ventral cords (e.g., annelids, arthropods), nerve net-like in hemichordates, and do not resemble those of higher chordates that are organized around a dorsal “hollow tube” (see for example review in Moroz, 2009). To facilitate comparison and to favor the identification of “robust homology hypotheses” Richter et al. (2010) proposed a neuroanatomical terminology of invertebrate nervous systems. We will not necessarily follow the neuroanatomical terminology adopted by Richter et al. (2010) since we will prefer to refer to the classic terms as defined by Young and coworkers for cephalopod brains (Young, 1971; review in Nixon and Young, 2003).

In several protostomes, such as annelids and insects, the ‘higher’ centers (here considered as centers of associative and high-order sensory/motor neural-processing), such as the mushroom bodies, tend to congregate in anterior nervous territories, similar to the situation that occurs in the vertebrate pallium (Arendt, 2008; Loesel and Heuer, 2010; Tomer et al., 2010; Wiersma and Roach, 2011). In each of these taxa, ‘higher’ neural-centers are found in a few species, but absent in more ’basal’ species of the group, suggesting that complex brains and higher centers evolve as a consequence of an independent specialization (Farris, 2008; Hejnol and Martindale, 2008; Moroz, 2009). An alternative explanation is that these species share molecular machinery with their deep ancestries, and that the ‘loss’ of higher centers in the basal species is the result of secondary simplification (Tomer et al., 2010). Neural structures such as the spinal cord, the hypothalamus, and basal ganglia have their ‘equivalents’ in annelids (Denes et al., 2007; Tessmar-Raible et al., 2007) and insects (Arendt and Nubler-Jung, 1999; Loesel et al., 2002; Strausfeld and Hirth, 2013) and are considered to share common molecular and structural profiles.

Molluscs allow an exploration of the potential evolutionary scenarios of nervous system evolution, due to the variety of different organizations (review in Kandel, 1979) of their acephalic ganglia, simple medullary cords, and centralized brains (Bullock, 1965a,b,c,d) that appear to be dissimilar to those of insects and vertebrates (Budelmann, 1995; Budelmann et al., 1997; Hochner, 2010). Molluscs also provide examples where some independent parallel events of centralization of nervous systems occur (Moroz, 2009).

Within the phylum Mollusca the coleoid cephalopod *Octopus vulgaris* has an exceptionally large brain that includes more than 30 differentiated lobes (Young, 1971), numerous cells (Young, 1963) possibly belonging to different cellular-types (Young, 1932; Bogoraze and Cazal, 1944; Young, 1972), highly organized neuropils and fasciculated tract bundles (Young, 1971; Hochner et al., 2006).

Here we review recent molecular, anatomical and developmental data to explore possible “homologies” of cephalopod neural structures with respect to vertebrate brains, a challenging task considering the more than 500 million years of independent evolution (see for example: Packard, 1972; Kröger et al., 2011; Roth, 2013). It is without doubt that many sensory-motor systems, locomotor abilities, and behaviors of cephalopods are traceable into vertebrate equivalents (e.g., Budelmann, 1995; Budelmann et al., 1997; Hochner and Glanzman, 2016; Villanueva et al., 2017). It is also true that the cephalopod brain is “truly molluscan” in its anatomical organization, making attempts to draw parallels between more than 30 lobes identified in its ‘central’ nervous system “and the brains of vertebrate species unrealistic” (Packard, 1972; see also Shigeno et al., 2015). However, some very “striking resemblances” occur (Packard, 1972): (i) the deep retina of fish and the surface of cephalopod optic lobe, (ii) the neural-architecture of the peduncle lobe in the octopus brain (which recalls the folia arrangements of the vertebrate cerebellum), (iii) the vertical lobe which is considered the analog of the mammalian limbic lobe (Young, 1991, 1995).

We summarize classic and modern views regarding neural-functional equivalencies between cephalopods and vertebrates, and highlight additional insights emerging from recent molecular and neurophysiological studies. Furthermore, we outline an embryological approach that allows identification of some features of relevance to the evolutionary paths leading to the neural centralization and differentiation of the cephalopod brain (see also: Focareta et al., 2014; Wollesen et al., 2014, 2015a; Shigeno et al., 2015; Buresi et al., 2016).

### The ‘Brain’ of Cephalopods – An Outline and a Summary of Novelties

**FIGURE 1:**
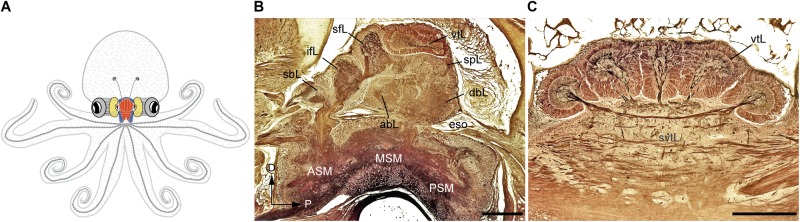
The adult *Octopus vulgaris* brain. **(A)** Schematic outline of octopus body and the relative relationships to the main components of its nervous system. **(B)** A longitudinal section of the supra- and sub-esophageal mass of *O. vulgaris* (parasagittal plane). **(C)** A cross section of the vertical lobe (supra-esophageal mass), showing the five distinct gyri. The esophagus lies at the center between the supraesophageal and subesophageal mass. Sections of stained with the Cajal silver method. abL, anterior basal lobe; ASM, anterior subesophageal mass; dbL, dorsal basal lobe; eso, esophagus; ifL, inferior frontal lobe; MSM, middle subesophageal mass; PSM, posterior subesophageal mass; sbL, superior buccal lobe; sfL, superior frontal lobe; spL, subpedunculate lobe; svtL, subvertical lobe; vtL, vertical lobe. Scale bars: 500 μm.

In the octopus, as far other cephalopod species, the ‘brain’ is assembled through a series of ganglia of molluscan origin to form lobes that are fused together into masses (for the common octopus see description in Young, 1971; see also an outline of the brain and its main connections in **Figure 1A**). These are connected to periphery by many nerve trunks regulating the arms, viscera and other part of the animal’s body connecting with the sub-esophageal mass (SUB; lower structure in **Figure 1B**), and which in turn connects directly or indirectly to the lobes of the supra-esophageal mass (SEM; **Figure 1B**, top).

The major connectives, commissures, and matrix of interneurons have been analyzed extensively using the Golgi and Cajal reduced silver staining methods (Young, 1971; see also **Figures 1B,C**). In addition, horseradish peroxidase, cobalt, and carbocyanine dye tracing methods have provided further detail (e.g., Young, 1971; Saidel, 1982; Budelmann and Young, 1985; Plän, 1987; Robertson et al., 1993).

According to the classical view, the SEM, lying above the esophagus, is dorsal with respect to the body-axis, while the SUB, extending below the esophagus, is ventral. The foremost dorsal structure of the SEM, the vertical lobe (vtL in **Figures 1B,C**), is considered one of the most distinctive structures in the cephalopod brain. It comprises about 14% of the volume of the entire supra-esophageal mass in an adult octopus (Frösch, 1971; Maddock and Young, 1987), and has over 25 million nerve cells, more than half of all the cells located in the supraesophageal mass (Young, 1963). When considered with the nearby center, i.e., the superior-frontal lobe (sFL in **Figure 1B**), the ‘vertical lobe system’ is recognized as the largest learning and memory (‘higher’) center among all known invertebrate neural structures (Young, 1991; Shomrat et al., 2015; Marini et al., 2017; Turchetti-Maia et al., 2017).

The dorso-ventral orientation of the brain with respect to the body-axis, as described above (see also **Figure 1**) seems unconfirmed by developmental studies. The antero-posterior expression of *Hox* genes (a family of transcription factors responsible for defining axial identity in bilaterians, Pearson et al., 2005) in structures such as the cephalopod brachial and buccal crown, funnel, and stellate ganglia are not predicted by *Hox* collinearity. Their expression along the axis does not appear to demonstrate the canonical nested domains characteristic of these transcription factors (see Lee et al., 2003). Furthermore, as defined by embryological orientation along the body axis (see for example: Shigeno et al., 2008, 2010; Buresi et al., 2016), the brain areas controlling arms and brachial centers are considered ventral, while those controlling the mantle and visceral organs appear dorsal: a remarkable shift.

Despite some initial interest, the phylogenetic origins of cephalopod neural centers remain largely unexplored (Young, 1977a; Nixon and Young, 2003; see also discussion in Grasso and Basil, 2009). The recent genomic sequencing of *O. bimaculoides* (Albertin et al., 2015) and the possible availability of other cephalopod genomes in the near future opens a new era. The analysis of *O. bimaculoides* genome revealed that the basic neuronal gene repertoire of cephalopods is shared with that of many other invertebrates. However, the octopus genome appears to be characterized by extensive expansion of transposons and other gene families, including an unusual (for invertebrates) expansion in the protocadherins and the C2H2 superfamily of zinc-finger transcription factors (Albertin et al., 2015). These genome level novelties are rendered more complex by the already well established extensive RNA editing, particularly in nervous system cells, which allows diversification of the proteins that the cells can produce (Garrett and Rosenthal, 2012a,b; Liscovitch-Brauer et al., 2017).

A short list of cephalopod novelties, excluding a discussion on the *Bauplan*, may include: (i) an extraordinarily large cadherin gene encoding over 70 extracellular cadherin domains found to be highly expressed in octopus suckers; (ii) gene families expansions (e.g., protocadherins, zinc finger proteins, interleukin-17 like genes, G-protein coupled receptors, chitinases and sialines); (iii) novel octopus-specific genes expressed in specialized structures such as skin and brain; (iv) Vascular Endothelial Growth Factor (VEGF) pathway, a possible prerequisite for the development of a closed vascular system; (v) octopressin/cephalotocin co-occurrence, never before reported in invertebrates; (vi) horizontal gene transfer as a possible origin of reflectin gene, allowing dynamic iridescence and structural color change in the skin, in cephalopod clades (Albertin et al., 2015; Guan et al., 2017; Wang and Ragsdale, 2017). These may originate by increase in genome complexity in the clade linked to polyploidy, differential arrangements of key genes (e.g., *Hox* appearing not clustered), exceptional RNA editing capacities, expansion of transposable elements (e.g., Packard and Albergoni, 1970; De Marianis et al., 1979; Lee et al., 2003; Albertin et al., 2015; Liscovitch-Brauer et al., 2017).

## The Vertebrate-Like Neural Systems in Cephalopods

It is without doubt that the most classic examples of vertebrate/mammalian-like comparison of cephalopod brain-functioning is provided by the work of Young (1961, 1964, 1965b, 1976a, 1991, 1995) and Hobbs and Young (1973).

The parallelism is seen in different structures and functional analogies; these differences encouraged later authors to consider cephalopod brains as unfamiliar structures, when compared to bird and mammalian brains, and as examples of analogous functions worth exploring as examples of phyletic boundaries of consciousness (Edelman and Seth, 2009).

### Evolutionarily Conserved Axes as Defined by the Developmental Framework

**FIGURE 2:**
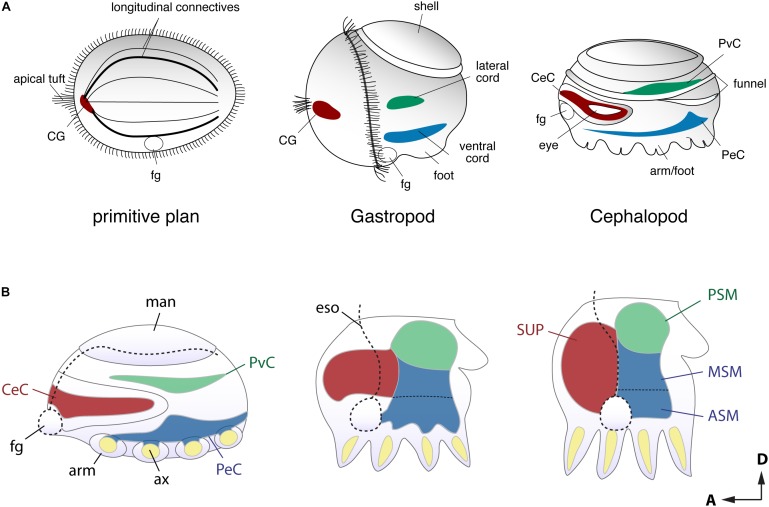
Comparison of the early stage embryonic nervous systems in three invertebrates. **(A)** Acoelomorph or planula-like larva (left), a gastropod veliger larva (middle), and nautiloid embryo (right), defining the comparable topography of neural patterns (modified after permission of Tokai University Press following Shigeno et al., 2010). The cerebral-, ventral- and laterally situated neural cords are shaded in red, green and blue, respectively. Due to their diffuse nature, the homology of these nerve cords remains unclear, but the putative ancestral condition is shown for comparison. **(B)** Schematic drawing of embryonic brain development in *O. bimaculoides* (inspired to information contained in Shigeno et al., 2015), showing a transition from medullary cords to a centralized brain. The foregut or mouth (fg) initially lies at the anterior, but it moves to a more ventral position at the later stage. Reference to the A–P and D–V axes are provided. ASM, anterior sub-esophageal mass; ax, arm axial cord; CeC, cerebral cord; CG, cerebral ganglion; eso, esophagus; fg, foregut or mouth; man, mantle; MSM, middle subesophageal mass; PeC, pedal cord; PSM, posterior subesophageal mass; PvC, palliovisceral cord; SUP, supraesophageal mass.

**FIGURE 3:**
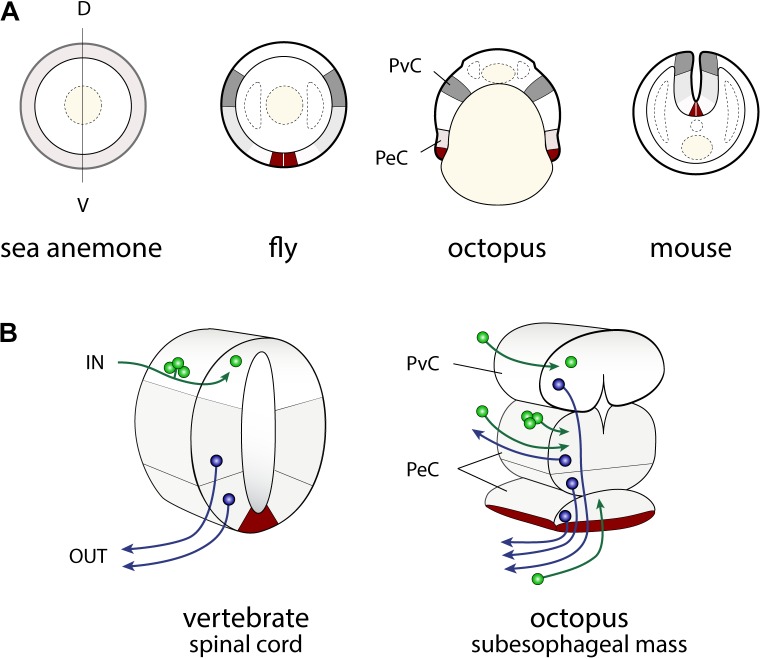
Similarities in the developmental plans of the vertebrate spinal cord and cephalopod sub-esophageal mass. **(A)** Comparison of the neurogenic territories along the embryonic dorso-ventral axes. The color codes indicate the candidates of comparable territories. **(B)** Oblique cross-dissected views of the vertebrate spinal cord and of octopus sub-esophageal mass. Red areas indicate the midline cells in the spinal cord, and possible comparable parts of the ventral position of the sub-esophageal mass. A dorso-ventral segregation pattern of input sensory (green) or output motor neurons (blue) exists in the spinal cord, while no such segregation is obvious in the octopus sub-esophageal mass (see the text for further explanation). PeC, pedal cord; PvC, palliovisceral cord; D, dorsal; V, ventral.

**FIGURE 4:**
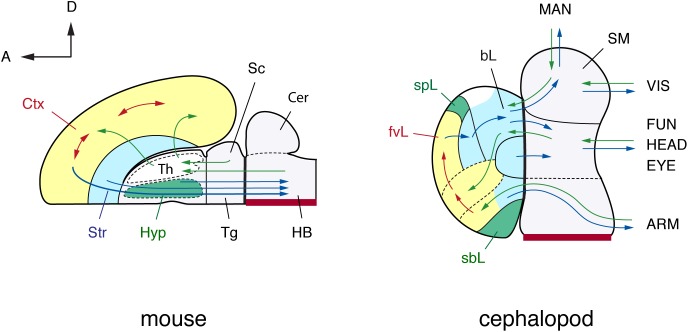
A brain-wide, flat-map comparison of the mouse and octopus brain. Topographical similarities are highlighted using color-coding. Note similarities with the pallium and basal ganglia, and neurosecretory centers (hypothalamus). The hypothalamus and the octopus neurosecretory systems differ superficially in adult brains with the neurovenous tissues (Young, 1970), considered neurosecretory areas in cephalopod brain, such as the para-vertical and the sub-pedunculate that are situated more laterally together with the optic lobes (omitted for simplification in this figure). The sensory inputs and motor outputs indicate functionally equivalent centers. The maps are arranged along the embryonic A–P and D–V axes (outline of mouse brain inspired by the information included in: Rubenstein et al., 1994, 1998; Puelles and Rubenstein, 2003; Swanson, 2007). Cephalopod embryonic brains are initially cord-like, and the topographic position of adult brain centers is traced back to embryological position via successive histological observation (Marquis, 1989; Shigeno et al., 2001, 2015). The main driver pathways (see text for details) are selected following Young (1971) and Plän (1987). (mouse): Cer, cerebellum; Ctx, cerebral cortex; HB, hindbrain; Hyp, hypothalamus; Sc, superior colliculus; Str, striatum; Th, thalamus; Tg, tegmentum; (cephalopod): ARM, arm nerve cord; bL, basal lobe; fvL, frontal and vertical lobe; EYE, eyes; FUN, funnel; HEAD, head; MAN, mantle; SM, subesophageal mass; sbL, superior buccal lobe; spL, sub-pedunculate lobe; VIS, visceral organs.

Developmental approaches have been used to probe how the complex brain centers and body parts developed during the evolutionary history of cephalopods (**Figure 2**). Embryological studies suggest that all molluscan nervous systems share an early developmental stage in which three neurogenic domains of the ganglia or medullary cords at the cerebral, ventral, and lateral position are present (Naef, 1928; Haszprunar, 1992; Shigeno et al., 2010, 2015; **Figure 2A**). These neural cords correspond to the cerebral, pedal, and palliovisceral ganglia (or cords), respectively (Marquis, 1989; Shigeno et al., 2015; **Figure 2B**). Based on topographical criteria and the neural composition (exemplified by the form of neurons and organization of tracts, for example) these may be compared to analogous structures in vertebrates such as the mammalian spinal cord (**Figure 3**) and fore- and mid-brains (**Figure 4**).

In particular, the dorso-ventral (D-V) neural arrangement of the cephalopod subesophageal mass may allow comparison with the medio-ventral parts of the vertebrate spinal cord; the ventral peripheral layer of cells of the subesophageal mass (see dark red in **Figures 3B, 4**) resembling the midline cells of the spinal cord, and most of the inputs (sensory) and outputs (motor) to/from the structures are conserved along their respective dorsal and ventral arrangements (**Figure 3B**).

Traditional terminology for the adult cephalopod brain distinguishes between the anterior and posterior parts of the subesophageal mass (Young, 1971). By contrast, the cephalopod embryological axis, as defined by Fioroni (1978), allows us to identify the antero-posterior (A–P) axis of the cephalopod body as corresponding to the D–V axis of vertebrates and thus allowing a comparison with the vertebrate spinal cord.

Developmental regulatory gene studies seem to support the cephalopod A–P/vertebrate D–V axis definition (see Lee et al., 2003). Recent molecular studies of various cephalopod species provide mixed evidence regarding the evolutionarily conserved nature of the axes. Tomarev et al. (1997) first found that a paired homeobox gene, *Pax-6*, is commonly expressed in the developing eyes and anterior cerebral fields of squid and vertebrate embryos. Along the A–P embryonic axis, the expression of the homeobox genes *otx*, *nkx2.1*, *hox*, and other homeodomain-containing genes, successfully distinguishes the developing brain fields (Lee et al., 2003; Buresi et al., 2012, 2016; Focareta et al., 2014). The *Pax2/5/8* expression domain has also been shown to mark a boundary between the A–P neural territories (Wollesen et al., 2015b), similar to those in the midbrain-hindbrain boundary of vertebrate brains. Furthermore, in cuttlefish embryos the D–V or medio-lateral axis expression domains of *pax6*-*pax2/5/8*-*pax3/7* genes successfully detect the topographically equivalent genes in the developing spinal cords of vertebrates (Buresi et al., 2016; see also Navet et al., 2017).

A number of other molecular studies involving neurogenic and signaling molecule genes have suggested evolutionarily conserved domains as well as ‘endemic’ novelties in the developing cephalopod brain (Baratte et al., 2007; Farfán et al., 2009; Navet et al., 2009; Ogura et al., 2013; Wollesen et al., 2014, 2015b; Yoshida et al., 2014; Shigeno et al., 2015; Focareta and Cole, 2016; Koenig et al., 2016).

### The Sensory and Motor Systems: The Spinal Cord and Hindbrain Analogy

The spinal cord is a principal sensory and motor center in vertebrate nervous systems (see **Figures 3, 4**). The dorsal neurons receive inputs from the sensory receptors, and the ventral motor neurons regulate motor actions, such as rhythmic movements of body muscles (e.g., Cohen et al., 1988; Grillner and Wallén, 1999; see also: Ayali et al., 2015; Berg et al., 2015) that are modulated by these inputs.

In an attempt to provide a possible comparative overview of vertebrate neural structures such as the spinal cord and the hindbrain and their putative cephalopod analogs we will consider below a few examples based on neural organization including somatotopy, dorso-ventral segregation of sensory- and motor-neural systems, peripheral vs. central neural domains, and fast escape responses in cephalopods.

#### Somatotopic Organization?

In the spinal cord, and in their invertebrate analog as for example in insects (e.g., Packard, 1884; Arendt and Nubler-Jung, 1999; Svidersky and Plotnikova, 2002), neurons are organized in columns with intrasegmental interneurons arranged functionally, representing a kind of somatotopic map (e.g., Butler and Hodos, 2005; Kiehn, 2016; Mantziaris et al., 2017).

In cephalopods somatotopic maps are considered not to exist (Zullo et al., 2009). In the higher motor centers such as the basal lobes (supra-esophageal mass), electrical stimulation has failed to identify any kind of somatomotor map, suggesting that there may be none in the cephalopod brain (Zullo et al., 2009; but see Gutnick et al., 2011), thus suggesting that cephalopods evolved a ‘unique’ solution for motor control (Gutnick et al., 2011; Hochner, 2012, 2013).

However, a somatotopic map has been suggested to occur in the sub-esophageal mass (e.g., Boycott, 1961; Monsell, 1980; Saidel, 1981; Dubas et al., 1986; Gaston and Tublitz, 2004; Gaston and Tublitz, 2006). A multi-color neuro-tracing study of the central distribution and the resulting three-dimensional arrangement of fin chromatophore motoneurons in the cuttlefish (Gaston and Tublitz, 2006), provided preliminary possible topographic organization of fin chromatophore motoneurons. These data support previous findings by Boycott (1961) who proposed a type of ‘somatotopy’ when considering the neural representation (in the chromatophore lobes, SUB) of chromatophores in the skin of the animals, depending on the species.

It is clear that the identification of segregated sensory- and motor-maps in cephalopod brains will require further studies.

#### Dorso-Ventral Segregation of Sensory-Motor Neural Systems

Along the D–V axis (as depicted above for cephalopod brain), centers characterizing the sub-esophageal mass and controlling specific body parts are arranged in the same order as those body parts: the pallial cavity, then the viscera, collar, funnel, head, ocular system, oculomotor system, and finally arms (Young, 1976a; Budelmann and Young, 1985; Gaston and Tublitz, 2006; **Figure 4**). However, and based on the available knowledge, neuronal segregation of the ventral motor and dorsal sensory neurons has not been reported for cephalopod sub-esophageal mass, and an analogy with the vertebrate arrangement seems difficult.

Despite differences (**Figure 3B**), in the octopus the great majority of inputs are collated in the dorsal- and mid-parts of the supra-esophageal mass (pedal and palliovisceral cords in **Figure 3B**), and most of the outputs project from the palliovisceral cord (ventral, **Figure 3B**), thus challenging a possible analogy with vertebrates. It is also true that the putative motor nerves projecting to the arms, as for the sensory information originating from the arms, usually come from both the ventral and dorsal sides of the SUB (Budelmann and Young, 1985). This is due to the arrangement of the anterior brachial lobes (SUB) with their intricate neuropil and connections, from where the four pairs of brachial nerves and the arm nerve cords originate (see description in: Young, 1971; Budelmann and Young, 1985; but see Lee et al., 2003).

#### Peripheral vs. Central Nervous System: The Case of the Arm Nerve Cord

Following Bullock (1965b), the foremost anterior part of the sub-esophageal mass accounts for “masses probably representing new ganglia associated with arms” (Bullock, 1965b, p. 1440) including the brachial ganglion (in the SUB) *sensu stricto*, the brachial nerves “to arms and suckers” and the interbrachial nerves (see also: Graziadei, 1971; Young, 1971). In the following pages, Bullock provided a description of the “complex nervous apparatus” characterizing arms and suckers as a “structure of the peripheral nervous system” (Bullock, 1965b; p. 1467, 1475–1479). It is interesting to note that Bogoraze and Cazal illustrated the central nervous system of the octopus including stellate ganglia and the related pallial nerves (see Figure 1 in Bogoraze and Cazal, 1944), a possible suggestion of an extended and distributed ‘central nervous system.’ It may be worth pointing out that the ‘brain’ is contained within the cranium (cf. skull) and that the ‘brain + spinal cord’ is in vertebrates considered as the central nervous system, as compared with the peripheral nervous system.

The overall arrangement of the arm nerve cord, medullary in the center with four small lateral cords, and its main features as “bilateral symmetry, segregation of tracts from synaptic regions, segmented outflow, and continuous medullary character of the axial cord” provides a strong analogy with “the vertebrate spinal cord; the similarity is increased on consideration of the physiological evidence of local, intersegmental, and superimposed higher mechanisms” (Bullock, 1965b; p. 1475).

The detailed descriptions provided by Graziadei, Young and coworkers (Graziadei, 1971; Young, 1971; Budelmann and Young, 1985; see also Margheri et al., 2011) are an example of a challenge for current neuroscience: to attribute a neural structure as complex as the arm nerve cord of octopods to the peripheral or to the central nervous system. Despite the typical invertebrate organization, we believe that the analogy with the vertebrate spinal cord is still largely unexplored, but likely.

Characterization of the acetylcholine synthetic enzyme choline acetyltransferase (ChAT) and serotonin in octopus arm nervous system supports this analogy. In the octopus arm two types of cholinergic nerves, cChAT-positive nerves from brain ganglia and pChAT-positive nerves intrinsic to the arm, have been identified (i.e., common type ChAT, cChAT; and peripheral type ChAT, pChAT; Sakaue et al., 2014). cChAT positive fibers appear in the arm ganglia and are likely related to brain efferents, appearing limited to fibers in octopus arm-nerve cord and in the cerebro-brachial tract. On the other hand, pChAT occurs in the intrinsic innervation of the octopus arm and is widely distributed in different nerve centers, probably associated with the sensory system (Sakaue et al., 2014). Similarly, two types of serotonin-like innervation have been shown in the arm: one type with fibers originating from the brain and innervating the periphery through the cerebro-brachial tract, and the other providing an intrinsic network to the cellular layer of the axial nerve cord (Bellier et al., 2017).

We reiterate here that the idea that the arm-nerve cord of cephalopods is not simply a neural structure belonging to the peripheral nervous system (as traditionally accounted, see for example Hochner, 2012), but a case of convergent evolution with functional and structural analogies existing between the vertebrate spinal cord and the octopus arm-nerve cord.

#### Neural Structures Controlling Fast Escape Responses in Cephalopods, a Case of Analogy With the Hindbrain?

Mauthner cells (Sillar, 2009) are one of the most historically notable motor systems for locomotory behavior reported in agnathans, teleost fish, and many amphibians. These are responsible for a rapid change in directionality and promote escape behavior (Fetcho, 1991; Korn and Faber, 2005). Mauthner neurons are characterized by a large neuronal cell, usually possessing a giant banana-shaped cell body located on either side of the midline in the brainstem with axon crossing to the contralateral spinal cord where they synapse with somato-motor neurons. The inputs to Mauthner neurons are primarily from receptors of the vestibular, auditory, and lateral line systems. In fish, for example, the neurons are not the same size in all species and this is considered to be linked to differences in taxa and possibly habitat (Zottoli, 1978).

We consider a similar neural system being present in cephalopods: the giant fiber system of squid (e.g., *Doryteuthis* or *Loligo*) and the magnocellular lobe of cuttlefish and octopus. The activation of giant axons induces the rapid escape behavior and vigorous jet propulsion (Otis and Gilly, 1990). Like the Mauthner neurons, the giant fiber system of squid is composed by a series of cells, some of them reaching over 250 μm in diameter (in *Doryteuthis pealeii*, see Young, 1939; see also Young, 1976b). These giant cells are multipolar with extensive dendritic arborization (Young, 1939, 1976b), resembling vertebrate neurons.

In squid, mantle contraction and jet propulsion are controlled by a giant fiber system consisting of two sets of three giant neurons organized in tandem (Young, 1939). According to J. Z. Young and later Authors, the “axons arising from the two first-order giant cells pass backward into the neuropil of the palliovisceral ganglion. Here they approach one another in the middle line, and are joined by the inter-axonic bridge […]. The interest of this remarkable structure is that in the adult it consists not of a chiasma or crossing of two distinct fibers, but of a true protoplasmic bridge” (Young, 1939, p. 477). Such an organization allows synaptic inputs from either side of the brain to be integrated and propagated down the giant fiber system as a symmetrical event for synchronous contraction of both sides of the mantle musculature (Pozzo-Miller et al., 1998). After the chiasm, these giant axons branch and establish synapses (chemical and electrotonic-gap synaptic junctions) with several second-order giant axons in the neuropil of the palliovisceral lobe (SUB). From these cells, axons project from the central palliovisceral lobe (SUB) to the stellate ganglion in the mantle via the pallial nerve forming the presynaptic elements at the giant synapses (Young, 1939; Martin and Miledi, 1986). The axons of the giant system of cephalopods are thus part of an intricate network with other regions of the brain (Young, 1939; see also: Young, 1977a; Nixon and Young, 2003).

In the brain of *Sepia officinalis* and *O. vulgaris* the magnocellular lobe serve the same function (Young, 1971; Chichery and Chanelet, 1976). Interestingly, differences in cellular sizes among different species exists; however, their preserved functions (i.e., neural control and initiation of fast locomotion and escape responses) indicate another possible analogy when comparing vertebrates (e.g., fish) and cephalopods (Young, 1977a; Zottoli, 1978).

Further studies are required to provide data to support or contradict this working hypothesis.

### The Neurosecretory System: An Analog to the Hypothalamus

Neurosecretion is pivotal for orchestrating essential body functions and metabolism and is considered a common metazoan phenomenon (Dorn, 1998; Hartenstein, 2006; Tessmar-Raible, 2007). Neurosecretory cells are characterized by large dense core vesicles that are not produced locally (at the synapse), but in the cell soma and have to travel along an axon (sometimes over a considerable distance) to reach their release site. In addition, neurosecretory centers are usually clustered in specific areas.

In vertebrates, the hypothalamus is located at the rostro-ventral region of the forebrain and among cellular-types are a set of neurosecretory cells (Butler and Hodos, 2005). The evolutionary origins of neurosecretory cells can probably be traced to a common bilaterian ancestor or pre-bilaterian animal such as a cnidarian (Hartenstein, 2006; De Velasco et al., 2007; Tessmar-Raible et al., 2007).

The neurosecretory centers of molluscan nervous systems tend to be distributed in the cerebral ganglia (gastropods). Alternatively the cells tend to be organized into distinct clusters in the preoral regions associated with the esophagus, or the stomatogastric nervous systems (e.g., Simpson et al., 1966; Kandel and Kupfermann, 1970). In cephalopods, neurosecretory cells are mainly found in the buccal (SEM), sub-pedunculate (SEM), and in part of dorsal basal lobes again in the supra-esophageal mass (Young, 1970). Surrounding the ‘brain’ there are several other ‘potential’ neurosecretory regions such as those present in the sub-buccal and sub-pedunculate areas and in the optic gland, and the neurovenous tissue of the vena cava (Bogoraze and Cazal, 1946; Young, 1970).

The optic glands and the sub-pedunculate lobe are considered to function as neurosecretory centers related to reproduction and are the candidates for pituitary-hypothalamus analogs in the cephalopod brain (Wells and Wells, 1969). We would expect to see an analogically equivalent area in the vertebrate brain, and indeed, studies have detected in the above-mentioned cephalopod brain centers a subset of neurons containing hypothalamus abundant molecules such as GnRH (Di Cosmo and Di Cristo, 1998; Iwakoshi-Ukena et al., 2004; Kanda et al., 2006; Shigeno and Ragsdale, 2015) and duplicated vasopressin orthologs, octopressin and cephalotocin (Kanda et al., 2003a,b, 2005; Minakata, 2010; Shigeno and Ragsdale, 2015). Unfortunately, it is largely unknown how each neurosecretory tissue is derived from those of molluscan ancestors and what its relationship is to other higher brain centers.

In any case neurosecretion is a common control mechanism and cephalopods and vertebrates both show discrete groups of neurons in their ‘brain’ that secrete peptides with an action at a distant site via the blood. Note that we are not proposing that specialized neurosecretory areas are unique to cephalopods and vertebrates, as they are present in most animal species studied to date (Hartenstein, 2006; Tessmar-Raible, 2007; Williams et al., 2017).

### Higher Sensory Centers: An Analog to the Thalamus

To the best of our knowledge, a cephalopod equivalent of the vertebrate thalamus has not been proposed. The thalamus is often referred to as a sensory relay center though which almost all sensory inputs run on their way to the cerebral cortex or pallium (Riss et al., 1972; Swanson, 2007). It is a gatekeeper to the cortex and is considered to have a role in ‘pain’ and ‘consciousness’ (Alkire et al., 2008; Schiff, 2008; Baliki and Apkarian, 2015; Rajneesh and Bolash, 2018). It is composed of a number of nuclei that usually have distinct sensory fields.

Using the above features as a basis for comparison we suggest that in the cephalopods dorsal basal- and sub-vertical lobes could be considered as candidates for analogs to the vertebrate thalamus.

The dorsal basal and sub-vertical lobes receive many input fibers from the entire body via direct and indirect pathways from the sub-esophageal mass (Young, 1971), suggesting that it is a relay center for the ‘cortically located’ frontal and vertical lobes in cephalopod brain. We counted between 11 and 15 main tracts originating and/or departing from (afferent and efferent) the two structures, i.e., dorsal basal- and sub-vertical lobes, based on the description available for *O. vulgaris* (Young, 1971); an estimation of the number of neural fibers composing these tracts is not available, or only possible for part of the dorsal basal following Plän (1987). The dorsal basal lobe also provides many outputs to the lower motor centers, suggesting it can also be categorized as a higher or intermediate motor centers (Boycott, 1961; Zullo et al., 2009). It is without doubt that the connectivity of these centers is very extensive, thus supporting our view of that they are relay centers analogous with the thalamus in vertebrates.

The inferior frontal lobe also appears to be another candidate. It is a major chemo-tactile sensory-motor center processing information originating from the suckers and arms, just as occurs in the olfactory cortex. It is involved in learning and memory recall being part of the so-called chemo-tactile memory system (Wells, 1959; Young, 1995). Also in this case, Young (1971) describes four afferent and seven efferent connections to/from the inferior frontal lobe, and considers it as the main part of the matrices involved in the chemo-tactile sensory-motor learning system (Young, 1991, 1995).

The above account is mainly based on *O. vulgaris*. In our view, a comparative analysis including information on main connections of homologous structures in the brain of other cephalopod species may provide further insight (for cephalopods – Decapodiformes, see: Young, 1974, 1976b, 1977b, 1979; Messenger, 1979; for a vertebrate based comparative overview see Butler, 2008).

### Higher Motor Centers: Analogs to the Basal Ganglia

In vertebrates, the higher motor centers receive sensory inputs and modulate their output to the pattern generators, located in “lower” parts of the central nervous system, to orchestrate the actions of multiple appendages to regulate posture, orientation, breathing, autonomic control of the viscera, and also habit formation (Reiner et al., 1998; Yin and Knowlton, 2006). The basal ganglia and the dorsal striato-pallial complex along with the spinal cord, midbrain and cerebellum, are the major centers regulating the outputs of cascading projection neurons.

In different bilaterians the putative higher motor centers have been identified with different terminology (e.g., Young, 1971; Orrhage, 1995; Loesel et al., 2002; Strausfeld et al., 2006; Homberg, 2008; Beckers et al., 2011; Pfeiffer and Homberg, 2014): central complex (insects), arch-like bodies and midline neuropils (non-insect arthropods, annelids), cerebral commissures (other protostomes), basal lobe system (cephalopods). The homology of these structures among phyla remains uncertain, and each motor center has become independently specialized to the demands of each animal lineage, resulting in different body plans, locomotory systems, and life styles across these taxa.

Despite such specialization, it is possible that higher motor centers share a common origin that can be traced back to the cerebral or preoral commissural region of a bilaterian ancestor, since almost all bilaterian nervous systems, including primitive acoelomorphs, have several thick commissural pathways connecting paired cerebral ganglia with bilateral body parts (see description in Bullock, 1965a,b,c,d; see also Reichert and Simeone, 2001).

Just as in many vertebrate species, the higher motor centers of coleoid cephalopods are complex neural structures (Young, 1971, 1977b). The main motor output centers are the basal lobes in the supra-esophageal mass (Boycott, 1961). Based on neural connectivity and experiments testing function after lesion of specific areas of the cuttlefish ‘brain,’ the cephalopod anterior basal lobes have been proposed as being analogous to the vertebrate basal ganglia (Chichery and Chichery, 1987; Gleadall, 1990).

The anterior basal lobe and the vertebrate basal ganglia are both situated at the pre-oral and peri-esophageal regions at the base of the anterior brain, respectively (**Figure 4**; see also **Figure 1B**). Likewise, the major connectivity of the lobe and its functional structure are similarly hierarchical, progressing from motor pattern learning to central pattern controllers, initiators, generators, and motor neuron pools, and finally to behavior as is thought to occur in vertebrate brains (Stocco et al., 2010). Unfortunately, the physiological function of the basal lobes in cephalopods remains only vaguely known (Zullo et al., 2009) and so this hypothesis requires further testing.

It is noteworthy to mention that few studies maybe claimed in support of the existence (or not) of Central Pattern Generators (CPG) in cephalopods. We refer here to: (i) the excitable receptor units in the mantle of octopus by Gray (1960) and the neural control of breathing, that may provide indirect evidence for CPG; (ii) the tentacle strike of cuttlefish and squid, but with almost no data on neural control; (iii) the locomotor patterns involved in octopus crawling, with evidence that is difficult to interpret as CPGs *sensu stricto* (Levy et al., 2015; Levy and Hochner, 2017).

### The Peduncle Lobe: Analog of the Cerebellum

The cerebellum is involved in controlling balance, proprioception, and ocular reflexes via fixation on a target object, planning bodily movements and also motor learning. It is highly interconnected with the optic tectum, thalamus, and midbrain (Swanson, 2007).

The cephalopod peduncle lobe is a candidate analog for the cerebellum (Messenger, 1967a,b; Hobbs and Young, 1973; Young, 1976a; Messenger, 1979; Camm et al., 1985). According to the ultrastructural characterization of the peduncle lobe of *O. vulgaris* made by Woodhams (1977), and based on evidence about the effects on locomotor responses of the animal after lesions to this lobe, Woodhams (1977) suggested a close functional and morphological analogy to a folium of the vertebrate cerebellum. The presence of a conspicuous and characteristic array of parallel fibers, originating from the spine cells, in the neuropil of the lobe and their “striking resemblance to those of vertebrate cerebellar granule cells,” and “serial synaptic relays present along their length” support this conclusion (Woodhams, 1977, p. 329).

Like vertebrates, cephalopods have a hierarchical series of motor control centers that coordinates signals from the vestibular organs, eyes, and body (Young, 1976a). The fibers from the optic lobe run into the peduncle lobe along with those from the anterior basal and the magnocellular lobes, and then their outputs connect to the oculomotor center, i.e., the lateral pedal lobe in the SUB (Budelmann and Young, 1985) as is the case in vertebrates represented by the medulla-cerebellum-midbrain axis regulating vestibulo-ocular reflexes.

### The Associative (or Auxiliary) Centers: Analogs of the Pallium/Cerebral Cortex

A number of studies have used an evolutionary perspective to postulate the ancestral form of the pallium/cerebral cortex in both vertebrates and invertebrates (e.g., annelid and insect ‘brains’; see for example, Tomer et al., 2010; Strausfeld, 2012).

In some cephalopods, such as *S. officinalis* and *O. vulgaris*, experimental evidence for sleeping, decision-making, discrimination learning and lateralization of the brain suggests that cephalopods possess a higher level of cognitive ability (Mather, 1995, 2008; Edelman and Seth, 2009; Edelman, 2011; Marini et al., 2017) thus leading to the hypothesis that these cognitive features require in cephalopods the equivalent of a cerebral cortex as in mammals (Edelman et al., 2005; Edelman and Seth, 2009; Roth, 2013).

Through extensive experimentation using ablation of various brain areas followed by behavioral assays the higher centers, i.e., the frontal- and vertical lobe systems, have been shown to be involved in tactile and visual memory processing (Maldonado, 1963a,b, 1965; Young, 1971, 1991, 1995). These include (i) numerous uniquely distributed small-size interneurons, called amacrine cells (Young, 1971, 1979), (ii) the presence of parallel running fibers, and (iii) reverberating circuitry across different lobes (Young, 1991, 1995).

These areas are also characterized by synaptic long-term potentiation, neurotransmitter function, and heterogeneity of neurochemical identity (Hochner et al., 2003; Shomrat et al., 2008, 2010, 2011; Shigeno and Ragsdale, 2015; for review see: Shomrat et al., 2015; Turchetti-Maia et al., 2017). The reason for the deep homology between the vertebrate pallium and the cephalopod vertical lobe system – whether derived from a common ancestral plan or convergently evolved – remains uncertain, but the cephalopod vertical lobe is the best candidate for vertebrate pallium analog within the molluscan lineage (Young, 1991, 1995).

## Models for Associative Neural Networks

If functional equivalents of the cerebral cortex evolved independently in both the cephalopod and vertebrate brains, what is the common structural and/or functional principle that drove this? Here, we summarize the most likely hypotheses.

### The Paired Centers and Matrix Model

Young (1965b, 1991, 1995) studied the multi-level control of attack or retreat behavior resulting from the association of taste, touch, vision, and possible pain in response to the experience that animals have when interacting with objects or prey. According to Young, the ‘paired cortical centers,’ i.e., the inferior- and superior frontal-vertical lobe systems, determine the probability of a positive or negative response for pursuing a given ‘food items’. The systems are composed of combined matrices of axons with intersecting axes where memory is stored. The ‘classifying’ neurons of lower centers send signals to higher cortical lobes or take a short-cut by directly dictating the proper response to output motor neurons. In the ‘cortical’ centers (e.g., the median frontal lobe) the response is modulated to increase the probability of attack, unless this action is vetoed by the vertical lobe. This constructs a hierarchical system of decision-making as suggested by the selective theory of the vertebrate higher sensory centers such as cerebral cortex or cerebellum (Eccles, 1977; Edelman, 1978).

Unfortunately, this model has not yielded a hypothesis as to how the neural connectivity patterns and cell types are equivalent to those of vertebrate cerebral cortex.

### The Associative Learning Model

As reviewed by Marini et al. (2017), Young and coworkers were able to provide an associative learning model of the octopus brain (Boycott and Young, 1955; Maldonado, 1963c; Young, 1964, 1965b, 1991; Maldonado, 1965) based on the existence of a series of matrices (see also above) that allow computation and that were considered analogous to the limbic lobe of higher vertebrates (Young, 1991, 1995; review in: Borrelli and Fiorito, 2008; Marini et al., 2017). In the octopus ‘learning system,’ the small interstitial neurons (amacrines) and their synapses play an important role in learning by means of their sensitizing effects on reward and punishment signals coming from outside. This model explains the short fluctuations in memory recall and long-term cumulative changes via Hebb’s synaptic law, that frequent stimulation of certain synapses strengthen their signals and connectivity (Hebb, 1949). The associative learning of *O. vulgaris* has been also a ‘model’ in cybernetics (Clymer, 1973; Myers, 1992), and appears surprisingly similar to a more recent one, suggested for the learning system of insects (i.e., the mushroom bodies), particularly of the honeybee (Heisenberg, 2003). In the insects, the model posits on the assumption that sensory odor signals are spatio-temporally represented by synaptic sets of small intrinsic interneurons (Kenyon cells) in the neuropil of the mushroom body. The reward- or punishment-conditioned stimulus of these Kenyon cells strengthens synapses with their outputs.

**FIGURE 5:**
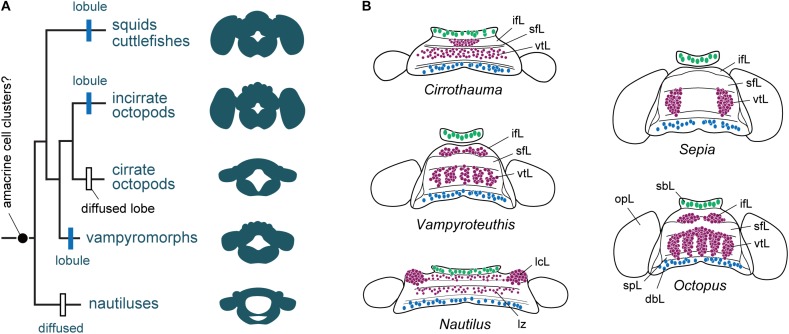
The evolution of cortical territories represented by a zonation in cephalopod brain evolution. **(A)** Phylogram of the evolution of brain complexity and emergence (still controversial) and organization of the amacrine cells into clusters. Based on the information included in Lindgren et al. (2012), and data assembled from Young (1965a, 1977a), Nixon and Young (2003). The centers are primitively zonal or band-like (*Nautilus*) and they are enlarged, or centralized or reduced in more ‘evolved’ species such as cuttlefish and octopus. **(B)** Homology of cell types and appearance of amacrine cells or their equivalent cell types (purple) in different cephalopod species. Homology of cell types in *Nautilus* is also controversial when compared with other taxa, but the gross similarity of topographical distribution is apparent. Large cells (green) are commonly localized in the buccal lobe area, which are often serotonergic (Wollesen et al., 2012). Toward the posterior end of the dorsal basal lobe clusters of GABAergic cells (blue) have been identified in octopus (Cornwell et al., 1993; Ponte, 2012). Outline of supra-esophageal mass and optic lobes are exemplified as a view from top; the overall shape of the brains is simplified as that of later embryonic stage. dbL, dorsal basal lobe; ifL, inferior frontal lobe; lcL, lateral cerebral lobe; lz, laminated zone of cerebral cord; opL, optic lobe; sbL, superior buccal lobe; sfL, superior frontal lobe; spL, sub-pedunculate lobe; vtL, vertical lobe.

Thus, octopus amacrine cells (**Figure 5**) and honeybee Keynon cells, as well as the octopus sub-vertical lobe and insect premotor centers, are candidates for being functionally equivalent analogs (Hochner, 2010). A partial cellular test of the system of functioning of the circuit underlying this ‘model’ has been achieved with the octopus (and cuttlefish) brain slice preparation (Hochner et al., 2003; Shomrat et al., 2008, 2011, 2015; Turchetti-Maia et al., 2017).

### The Reverberating Circuitry Model (Young, 1991, 1995)

The similarity in connectivity between the cephalopod superior frontal-vertical lobe system and the vertebrate hippocampal formation, based on matrices and reverberating feedback network structure (Maldonado, 1963a, 1965; Young, 1991), is the basis of this model.

Cephalopod learning capacity is not localized in certain layers or ‘grandmother cells’ but is distributed within a highly redundant series of matrices with recurrent circuits. Young emphasized the similarity with the hippocampal complex but avoided any clear statement about its relationship to the cerebral cortex (Young, 1991, 1995). Indeed, the existence of long term potentiation in the cephalopod vertical lobe (Hochner et al., 2003; Shomrat et al., 2008, 2011) maybe the basis of long term memory as it is considered in the hippocampus of vertebrates with minor molecular differences (Hochner et al., 2003; Turchetti-Maia et al., 2017). However, the higher matrix system of cephalopods is also comparable to that of the mammalian cerebral cortex which also forms distinct cellular and matrix units (Young, 1995).

### The Self-Organized Embodiment Model Without Somatotopy

The octopus higher motor centers are comparable to the motor cortex/pallium of vertebrates as a central control system, but they do not seem to be organized somatotopically (Zullo et al., 2009; Hochner, 2012, 2013). The lack of somatotopy in the higher motor centers of octopus may be explained by the non-biological concept of “self-organized embodiment” in robotics (Pfeifer et al., 2007; Cianchetti et al., 2012; Hochner, 2012; Laschi et al., 2012). The self-organized embodiment concept uses the dynamic interplay between the sensorimotor and a central controller to generate autonomous adaptive responses, and can explain very complex movements, such as the highly flexible motions of octopus arms.

Indeed, recent advances in artificial intelligence, including deep learning methods such as convolution networks (e.g., Mnih et al., 2015), show that neural-networks can be trained by and learn from numerically defined ‘weights’ provided to a whole network rather than from inputs due to local sensory representation. In support of this non-somatotopic idea, Grasso (2014) hypothesized that ‘higher’ neural centers of octopus have a role in time-series processing rather than acting as a spatial decoder. Reciprocal sensory information flow between the arms and ‘higher’ neural centers establishes a distributed memory trace in the Bayesian statistical sense. The reverberant circuits or recurrent matrices unique to the octopus frontal and vertical lobes produce signals lasting minutes to hours through Hebbian type learning. As a result, a brain-to-body spatial map or “Octo-munculus” (like the human “Homunculus”) would be depicted as information processing systems distributed throughout each arm and a brachial center in the brain (Grasso, 2014).

## Closing Remarks

Cephalopods are not the only invertebrates that exhibit sophisticated behavioral repertoire, higher-order learning and cognitive abilities (e.g., Avarguès-Weber and Giurfa, 2013; Giurfa, 2013; Perry et al., 2013; Marini et al., 2017; Mather and Dickel, 2017; van Duijn, 2017).

Here we attempted to overview available knowledge to propose a brain-wide comparative ‘model’ between cephalopod neural-systems and the neural structures characterizing vertebrates. Such a comparison identifies the cephalopod cerebral cord as analogous to the vertebrate forebrain and midbrain, and the pedal and palliovisceral cords in the cephalopod brain as being comparable to their putative equivalent in vertebrates: the spinal cord and the hindbrain (**Table 1**).

**Table 1:** A list of the higher sensory, motor, and neurosecretory centers in the ‘brains’ of cephalopods and vertebrates.

*Cephalopods*	*Vertebrates*
Cerebral cord	Fore- and midbrain
Frontal-vertical lobe	Cerebral cortex (pallium)
	Hippocampus
	Amygdaloid complex
Dorsal basal lobe	Thalamus
Anterior basal lobe	Basal ganglia
Buc^1^ and Spd^2^ lobes	Hypothalamus
Optic lobe	Tectum
Magnocellular lobe	Tegmentum
Peduncle lobe	Cerebellum
Pedal cord	Hindbrain and spinal cord
Palliovisceral cord	Hindbrain and spinal cord


*^1^Buc, buccal lobes, ^2^Spd, subpedunculate lobe. Data assembled from Bullock (1965b), Butler and Hodos (2005), Hartenstein (2006). See text for details and exceptions (e.g., oculomotor centers).*

The studies overviewed in this work have enabled us to draw functional analogies between cephalopod and vertebrate brains. Despite having fundamentally different anatomical organizations of adult brains, the embryologic patterns of longitudinal and transverse areas (orientation) along the A–P and D–V axes share similar topography in vertebrates and cephalopods. Surprisingly, the revised positional identities of the sub-esophageal centers (including brachial-, oculomotor-, funnel-, pallial- and visceral lobes) could account for much of the phylogenetic stability as well as novelties between the two taxa. Gene expression profiles controlling development support some of these proposed patterns, conserving the A–P and D–V axes of the brain and body regions as a whole (e.g., Albertin et al., 2015; Shigeno et al., 2015; Buresi et al., 2016; Navet et al., 2017).

Based on this developmental model, we have suggested that, unlike the vertebrate spinal cord, the octopus sub-esophageal system is arranged along the dorso-ventral body axis: the sensory-motor fibers run from the brachial, head, funnel, visceral mass, and the mantle. The basal lobes are placed, as in the basal ganglia, more anteriorly than the lower sensorimotor centers, and the associative centers (the frontal-vertical lobes) are at a more anterior-dorsal position as in the pallium or cerebral cortex of vertebrates. Our view establishes a topographical basis for a large-scale framework that encourages further discussion regarding analogs between the cerebral cortex, basal ganglia, and other vertebrate-like nervous systems in cephalopods.

## Author Contributions

SS conceived an earlier version of this manuscript. SS, PA, and GP provided an advanced draft. GF revised and finalized the manuscript. All authors discussed the content and commented the final manuscript. All authors read and approved the submitted manuscript.

## Conflict of Interest Statement

The authors declare that the research was conducted in the absence of any commercial or financial relationships that could be construed as a potential conflict of interest.
